# A New Electron Acceptor with *meta*‐Alkoxyphenyl Side Chain for Fullerene‐Free Polymer Solar Cells with 9.3% Efficiency

**DOI:** 10.1002/advs.201700152

**Published:** 2017-08-17

**Authors:** Zhenzhen Zhang, Liuliu Feng, Shutao Xu, Ye Liu, Hongjian Peng, Zhi‐Guo Zhang, Yongfang Li, Yingping Zou

**Affiliations:** ^1^ College of Chemistry and Chemical Engineering Central South University Changsha 410083 China; ^2^ Beijing National Laboratory for Molecular Sciences Institute of Chemistry Chinese Academy of Sciences Beijing 100190 China

**Keywords:** hexafluoroquinoxaline‐based polymers, m‐ITIC‐OR, *meta*‐alkoxyphenyl side chains, nonfullerene polymer solar cells

## Abstract

A new small molecule acceptor, m‐ITIC‐OR, based on indacenodithieno[3,2‐*b*]thiophene core with *meta*‐alkoxyphenyl side chains, is designed and synthesized. The m‐ITIC‐OR film shows broader and redshift absorption compared to its solution and matched energy levels with a hexafluoroquinoxaline‐based polymer donor‐HFQx‐T. Here, polymer solar cells (PSCs) by blending an HFQx‐T donor and an m‐ITIC‐OR acceptor as an active layer deliver the power conversion efficiency (PCE) of 6.36% without any posttreatment. The investigations demonstrate that the HFQx‐T:m‐ITIC‐OR blend films possess higher and more balanced charge mobility, negligible bimolecular recombination, and nanoscale interpenetrating morphology after thermal annealing (TA) treatment. Through a simple TA treatment at 150 °C for 5 min, an impressive PCE of 9.3% is obtained. This efficiency is among one of the highest PCEs for additive free PSCs. This is the first time alkoxyphenyl side chain is introduced into nonfullerene electron acceptor; more interestingly, the new electron acceptor (m‐ITIC‐OR) in this work shows a great potential for highly efficient photovoltaic properties.

## 1 Introduction

Exploitation and utilization of solar energy can not only solve the shortage of fossil fuels, the insecurity of the nuclear energy, and other energy problems, but also can push the development of economy and technology.^1^ Solution‐processed polymer solar cells (PSCs) are a promising way for the utility of solar energy, and have been intensively investigated due to their advantages of light weight, low‐cost, and flexible devices through roll‐to‐roll solution processes.^2^, ^3^, ^4^, ^5^ The PSCs based on conjugated polymers as an electron donor (D) and fullerene derivatives as an acceptor (A) have power conversion efficiencies (PCEs) of over 10%.^6^, ^7^, ^8^, ^9^, ^10^ However, the development of fullerene‐based PSCs is impeded by the shortcomings of the fullerene derivatives, such as weak absorption in the visible region and limited energy level modulation.

In recent two years, considerable efforts have been devoted to develop the nonfullerene acceptors. The organic/polymer electron acceptors based on perylene diimide (PDI) and naphthalene diimide (NDI) have shown a good performance as a result of the unique flat and rigid conjugated structure and modification.^11^, ^12^, ^13^, ^14^ Studies on the molecule self‐assembly of the traditional PDI and NDI derivatives show that they usually tend to form large crystalline domains^15^, ^16^; however, twisted PDI and NDI derivatives may decrease electron mobility.^17^, ^18^ Therefore, it is still a challenge to design PDI or NDI molecules with high electron mobility and nanoscale aggregated domain simultaneously.^19^ The other promising small molecules are based on rigid and coplanar indacenodithiophene (IDT) backbone, which exhibit strong and broad absorption as well as high charge carrier mobility. Moreover, the tetrahexylphenyl groups was attached to IDT, which can reduce intermolecular interactions and avoid strong self‐aggregation.^20^ Zhan and co‐workers reported a novel high‐performance acceptor named as “IEIC”, which is based on indaceno[1,2‐b:5,6‐b′]dithiophene and 2‐(3‐oxo‐2,3‐dihydroinden‐1‐ylidene)malononitrile(IC). When blended with a low‐bandgap donor polymer PTB7‐Th, the device showed an efficiency of 6.3%.^21^ Compared to IDT, a seven‐member fused ring indacenodithieno[3,2‐*b*]thiophene (IDTT) core with extended conjugation length can further improve light‐harvesting abilities and enhance charge mobilities.^22^ Zhan's group first reported IDTT‐based acceptor (ITIC), when blended with PTB7‐Th, the device delivered a PCE of 6.8%.^23^ Recently, using ITIC as electron acceptor, nonfullerene PSCs have reached high performance of 9–12%.^24^, ^25^, ^26^, ^27^, ^28^, ^29^ These promising results have attracted tremendous interests to investigate IEIC and ITIC derivatives as electron acceptors.

Among the ITIC derivatives, the side chain engineering of IDT or IDTT had drawn much attention to achieve high efficiencies, because the types of side chains play an important role in the energy level, mobility, morphology, and therefore PCEs.^30^, ^31^ For example, IC‐C6IDT‐IC with four *n*‐hexyl as side chains, exhibited a PCE of 8.71%,^32^ and ITIC‐Th with four 2‐thienyl groups showed a PCE of up to 9.6% with 1‐chloronaphthalene additive.^33^ However, from the reported ITIC derivatives, no attention was paid to using alkoxyphenyl replacing alkylphenyl of ITIC, indeed, alkoxyphenyl group seemed more easily synthesized via simple etherification, much lower cost, and more coplanar compared to its alkyl counterpart. The unique features of alkoxyphenyl group are beneficial for scalability and commercialization. Moreover, side chain isomerization influences the intermolecular self‐assembly of the conjugated structure due to steric effect and then have an impact on the performance of PSCs. It is noticed that metasubstitution of phenyl rings in donor materials has better solubility and good compatibility with PCBM.^34^, ^35^, ^36^ Recently, Li and co‐workers have reported an acceptor material m‐ITIC by replacing *para*‐alkylphenyl of ITIC with *meta*‐alkylphenyl; when m‐ITIC was blended with a polymer donor J61 after thermal annealing (TA) at 130 °C for 5 min, the PCE achieved was 11.77%. The performance is more superior than that of the J61/ITIC blend, because m‐ITIC with *meta*‐alkylphenyl instead of para‐alkyl‐phenyl side chains showed higher film absorption coefficient and higher electron mobility compared to its counterpart‐ITIC.^37^ Based on the above considerations, we first synthesized a new acceptor, namely, m‐ITIC‐OR, based on IDTT as a core with four *meta*‐alkoxyphenyl as side chains as well as IC as an end group. The new acceptor m‐ITIC‐OR possesses the following characteristics: (1) fused and planar ring from IDTT core can enhance absorption and charge transport; (2) four *meta*‐alkoxyphenyl side chains can ensure solubility in common solvents; and (3) IC as an end group leads to deep LUMO energy levels, which is appropriate to be an acceptor material.

**Figure 1:**
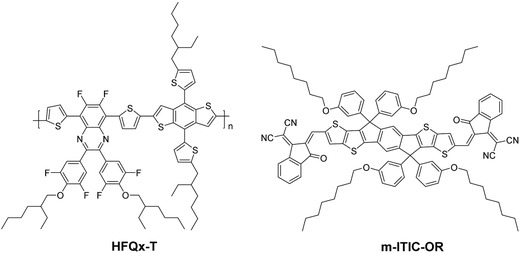
Chemical structures of polymer donor HFQx‐T and nonfullerene acceptor m‐ITIC‐OR.

To obtain high‐performance PSCs, the selection of donor materials with complementary absorption, suitable energy levels, and appropriate miscibility with acceptor materials to form nanoscale phase separation and bicontinuous interpenetrating networks is also critical.^38^, ^39^, ^40^ During the exploration of high‐performance donor materials, quinoxaline (Qx)‐based polymers attracted great attention due to the unique building block with interesting optoelectronic properties and easy modification.^41^, ^42^, ^43^ Very recently, we demonstrated a PCE of 9.12% for nonfullerene PSCs after additive and TA treatments with a tetrafluoroquinoxaline‐based polymer donor‐PffQx‐PS and ITIC as acceptor.^44^ Inspired by this successful strategy, most recently, our group synthesized a hexafluoroquinoxaline‐based polymer donor (HFQx‐T). The chemical structures of HFQx‐T and m‐ITIC‐OR are shown in **Figure** **1**a. Here, we fabricated devices based on HFQx‐T donor and m‐ITIC‐OR acceptor, which exhibited a PCE of 6.36% without any posttreatment. Interestingly, avoiding complicated additive process, only through a simple TA, the devices based on HFQx‐T:m‐ITIC‐OR blended films delivered a high PCE of up to 9.3% due to improved short‐circuit current density (*J*_sc_) and fill factor (FF), which is even higher than that of HFQx‐T:ITIC based devices. This efficiency is among one value of highest PCEs in additive‐free PSCs. The results indicate that *meta*‐alkoxyphenyl side chain is promising for constructing high‐performance electron acceptors. Combining its simplicity, low cost, and high performance, m‐ITIC‐OR shows a great potential for photovoltaic applications.

## 2 Results and Discussion

**Scheme 1:**
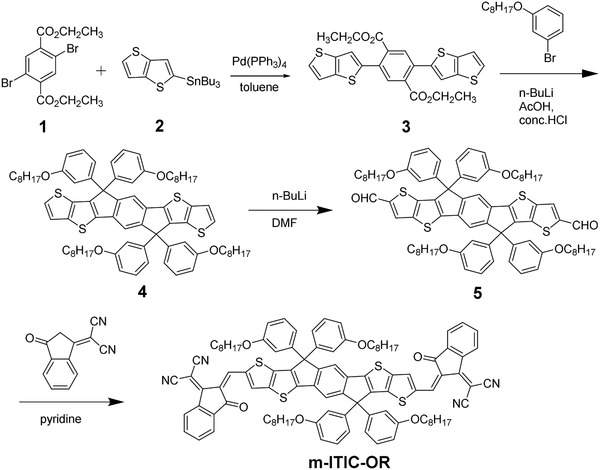
Synthetic route of m‐ITIC‐OR.

The detailed synthetic route of m‐ITIC‐OR is shown in **Scheme** **1**. The synthesis details are provided in the Supporting Information. Compound 3 was prepared by Stille cross‐coupling between 2,5‐dibromoterephthalic ethyl ester and tributyl(thieno[3,2‐*b*]thiophen‐2‐yl)stannane using Pd(PPh_3_)_4_ as the catalyst. Compound 4 was obtained by lithiation of 3‐(octyloxy)‐1‐bromobenzene and followed by reacting with compound 3, and then the crude product was refluxed in a mixture of acetic acid and concentrated hydrochloric acid for 4 h. Compound 4 was lithiated and then quenched with N,N‐Dimethylformamide (DMF) to obtain compound 5. Subsequent Knoevenagel condensation between compound 5 and 2‐(3‐oxo‐2,3‐dihydroinden‐1‐ylidene)‐malononitrile (IC) afforded m‐ITIC‐OR with 52% yield. All intermediates were characterized by ^1^H NMR, and the target product m‐ITIC‐OR was characterized by NMR and elemental analysis. NMR spectra of the m‐ITIC‐OR were shown in Figures S1 and S2 (Supporting Information). m‐ITIC‐OR shows good solubility in common solvents, such as chloroform (CF) and chlorobenzene (CB).

**Figure 2:**
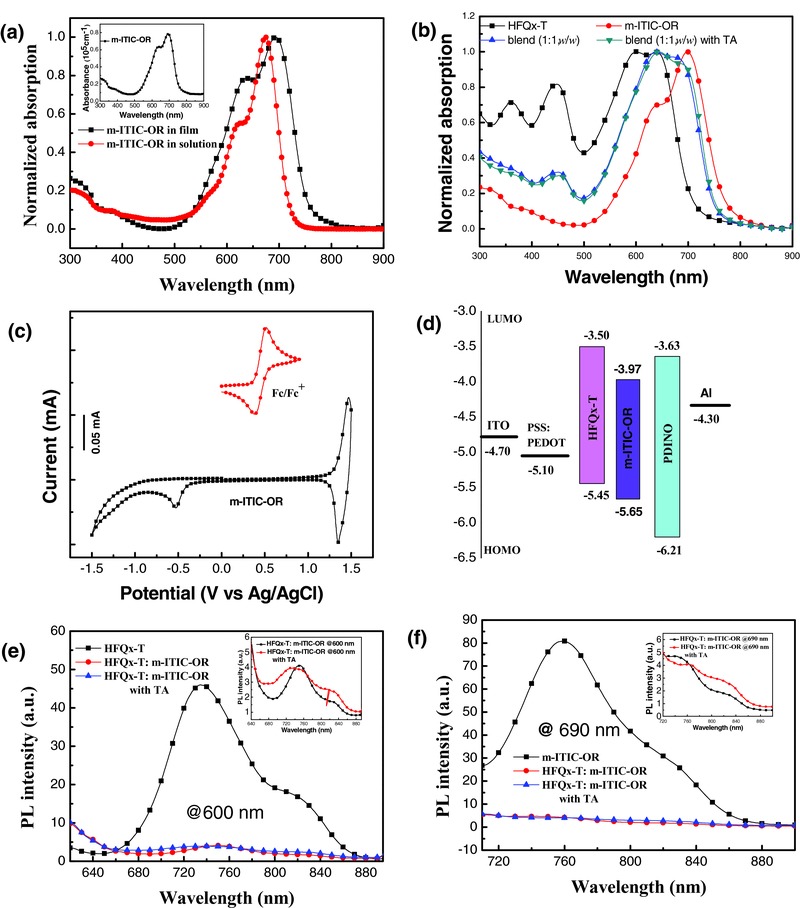
a) Solution and film absorption spectra of m‐ITIC‐OR; the inset shows the absorption coefficient of m‐ITIC‐OR in film state. b) Normalized absorption spectra of HFQx‐T, m‐ITIC‐OR, and HFQx‐T:m‐ITIC‐OR (1:1, w/w) blend films. c) Cyclic voltammograms of m‐ITIC‐OR in CH_3_CN/0.1M Bu_4_NPF_6_. d) Energy diagram relative to the vacuum level. e) PL spectra of pure HFQx‐T and HFQx‐T:m‐ITIC‐OR (1:1, w/w) blend films (excited at 600 nm); inset shows the PL spectra of blend films with and without TA (excited at 600 nm). f) PL spectra of pure m‐ITIC‐OR and HFQx‐T:m‐ITIC‐OR (1:1, w/w) blend films (excited at 690 nm); inset shows the PL spectra of blend films with and without TA (excited at 690 nm).

The UV–vis absorption spectra of pure m‐ITIC‐OR are shown in **Figure** **2**a. The m‐ITIC‐OR film exhibited broader absorption with a significant redshift of 36 nm compared to its solution state. The absorption maximum (λ_max_) of m‐ITIC‐OR film was at 690 nm with a maximum absorption coefficient of 7.8 × 10^4^ cm^−1^. m‐ITIC‐OR had absorption edge (λ_edge_) of 750 nm and the corresponding optical bandgap (*E*_g_^opt^ = 1240/λ_edge_) was 1.65 eV. As shown in Figure 2b, the λ_max_ and λ_edge_ of HFQx‐T were located at 604 and 705 nm, respectively. The HFQx‐T and m‐ITIC‐OR blend films before and after TA both provided a complementary absorption, covering a wide absorption range from 500 to 750 nm.

The electrochemical properties of HFQx‐T and m‐ITIC‐OR were measured by cyclic voltammetry (CV) with the ferrocene/ferrocenium (Fc/Fc^+^) redox couple (4.8 eV below the vacuum level) as an internal reference. As shown in Figure 2c, the HOMO energy levels (*E*_HOMO_) and LUMO energy levels (*E*_LUMO_) of m‐ITIC‐OR were estimated to be −5.65 and −3.97 eV from the onset oxidation and reduction potentials. The bandgap of m‐ITIC‐OR estimated from the CV was 1.68 eV, which was consistent with the optical bandgap. The *E*_HOMO_ and *E*_LUMO_ of HFQx‐T donor were also measured to be −5.45 and −3.50 eV, respectively (Figure S3, Supporting Information). The energy diagram relative to the vacuum level is shown in Figure 2d and the optical together with electrochemical data of m‐ITIC‐OR are summarized in **Table** **1**. Notably, the LUMO energy offset (Δ*E*_LUMO_) between HFQx‐T and m‐ITIC‐OR is 0.47 eV, which is sufficient for the exciton dissociation of the donor HFQx‐T and the electron transfer from HFQx‐T to m‐ITIC‐OR. Despite HOMO energy offset (Δ*E*_HOMO_) is only 0.20 eV, the hole transfer from the m‐ITIC‐OR acceptor to the HFQx‐T donor appeared to be relatively efficient, as can be seen from the photoluminescence (PL) quenching measurement. The PL spectra of the HFQx‐T (excited at 600 nm) and m‐ITIC‐OR (excited at 690 nm) films as well as the blend films of HFQx‐T:m‐ITIC‐OR (1:1, w/w) (excited at 600 and 900 nm) are shown in Figure 2e,f, the excitation wavelength was selected according to their maximum absorption. The pure HFQx‐T film exhibited a broad PL emission in the range of 680–860 nm, however, the PL emissions were quenched by 92% in the HFQx‐T:m‐ITIC‐OR blend films, which indicated efficient electron transfer from HFQx‐T to m‐ITIC‐OR. The PL intensities of m‐ITIC‐OR were also significantly quenched (95%) by HFQx‐T in the HFQx‐T:m‐ITIC‐OR blend films. The results provide clear evidence that efficient exciton separation and hole transfer can take place under low energy offset for nonfullerene PSCs. This efficient charge transfer is beneficial to obtaining a high *J*_sc_ in PSCs. Based on the above analysis, the HFQx‐T:m‐ITIC‐OR is expected to possess good photovoltaic performance. It has no prominent quenching efficient before and after TA treatment and even has stronger PL intensity with TA treatment in some wavelength range. The results indicate that TA treatment has little effect on charge transfer at the HFQx‐T and m‐ITIC‐OR interface. Although TA treatment is unfavorable for charge transfer, TA can induce microphase separation and form highly ordered interpenetrating networks as described below, which leads to an increase of *J*_sc_ and FF.^45^

**Table 1:** Optical and electrochemical data of m‐ITIC‐OR

	λ_max_	λ_edge_	*E*_g_^opt^	α	*E*_HOMO_	*E*_LUMO_	*E*_g_^cv^
Acceptor	[nm]a)	[nm]b)	[eV]c)	[cm^−1^]d)	[eV]	[eV]	[eV]e)
m‐ITIC‐OR	690	750	1.65	7.8 × 10^4^	−5.65	−3.97	1.68

^a)^ Absorption maximum of thin film

^b)^ Absorption edge of thin film

^c)^ Optical band gap calculated from the absorption edge of thin film: *E*_g_^opt^ = 1240/λ_edge_

^d)^ Absorption coefficient of films

^e)^ Electrochemical bandgap obtained from *E*_LUMO_ to *E*_HOMO_.

**Figure 3:**
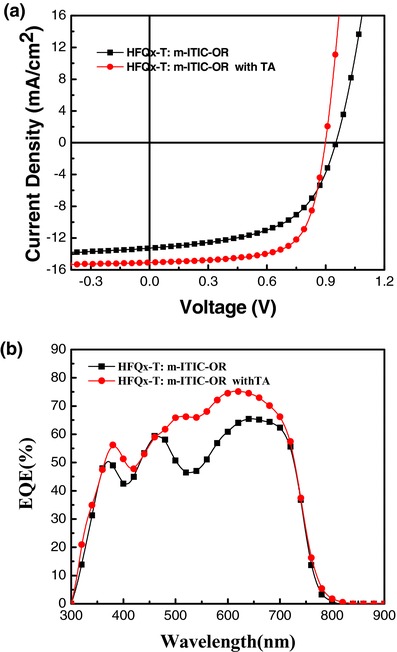
a) *J–V* curves of the PSCs based on HFQx‐T:m‐ITIC‐OR (1:1, w/w) with and without TA, under illumination of AM 1.5, 100 mW cm^−2^. b) EQE spectra of PSCs based on HFQx‐T:m‐ITIC‐OR (1:1, w/w) with and without TA.

To investigate photovoltaic applications of HFQx‐T:m‐ITIC‐OR blend films in PSCs, we fabricated PSCs with conventional structure of indium tin oxide (ITO)/poly(3,4‐ethylenedioxythiophene):poly(styrenesulfonate) (PEDOT:PSS)/active layer/PDI functionalized with amino N‐oxide (PDINO)/Al, where PEDOT:PSS and PDINO were hole and electron transport layer, respectively.^46^ The HFQx‐T:m‐ITIC‐OR weight ratio and annealing temperatures were well optimized. The detailed photovoltaic data are summarized in Table S1 (Supporting Information). The current‐voltage (*J–V*) curves of the optimized HFQx‐T:m‐ITIC‐OR weight ratio (1:1, w/w) under the illumination of AM 1.5G, 100 mW cm^−2^ are shown in **Figure** **3**a and corresponding photovoltaic parameters are listed in **Table** **2**. The PSCs based on HFQx‐T:m‐ITIC‐OR blend films showed a PCE of 6.36% with a *V*_oc_ of 0.95 V, a *J*_sc_ of 13.48 mA cm^−2^, and a FF of 0.50. With 1:1 D/A weight ratio, the PCE was further improved to 9.3% with a *V*_oc_ of 0.90 V, a higher *J*_sc_ of 16.15 mA cm^−2^, and a FF of 0.64 by TA treatment at 150 °C for 5 min, which is higher than 9.07% efficiency of HFQx‐T:ITIC based devices under optimized conditions. The results indicated that TA is an efficient posttreatment in HFQx‐T:m‐ITIC‐OR blend films and m‐ITIC‐OR has great prospect for photovoltaic applications. The corresponding external quantum efficiency (EQE) spectra of the devices with or without TA are shown in Figure 3b. The devices based on HFQx‐T:m‐ITIC‐OR blend films displayed a broad photo‐response from 300 to 800 nm. Obviously, the maximum EQE peak reached 76% after TA, while the maximum value was only 66% without TA, which indicated that the photo‐electron conversion efficiency of the HFQx‐T:m‐ITIC‐OR blend films via TA treatment was higher than that of blend films without TA. This may be the reason for higher *J*_sc_ after TA. *J*_sc_ values of the devices calculated from integration of the EQE spectra were 15.67 (TA) and 12.95 (without TA), in good agreement with the values from the *J–V* curves (within 5% mismatch), which suggested the results were reliable.

**Table 2:** The photovoltaic characteristics of the HFQx‐T:m‐ITIC‐OR blend films

Active layer	Ratio	*V*_oc_ [V]	*J*_sc_ [mA cm^−2^]	FF [%]	PCE [%]	Average PCE [%]c)
HFQx‐T:m‐ITIC‐OR	1:1a)	0.95	13.48	50	6.36	6.28
	1:1b)	0.90	16.15	64	9.30	9.13

^a)^ Without annealing

^b)^ Annealing at 150 °C for 5 min

^c)^ Average PCEs from ten devices.

**Figure 4:**
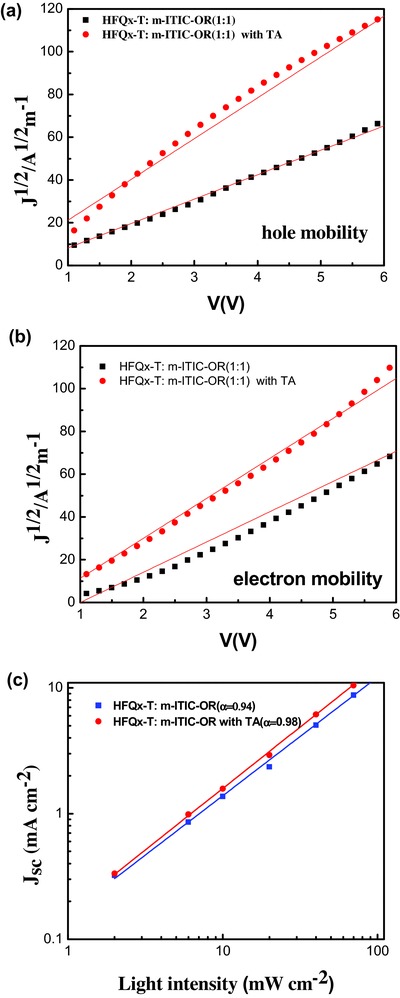
a) The hole mobilities of the HFOx‐T:m‐ITIC‐OR with or without TA. b) The electron mobilities of the HFOx‐T:m‐ITIC‐OR with or without TA. c) Light intensity dependence of *J*_sc_ of the PSCs.

To further understand the TA effects on the device performance, the charge transport properties of the HFQx‐T:m‐ITIC‐OR blends were investigated using space‐charge‐limited current (SCLC) method, and the results are shown in **Figure** **4**a,b, and the related data are summarized in **Table** **3**. The hole‐only and electron‐only devices were measured with devices structure of ITO/PEDOT:PSS/active layer/Au and ITO/ZnO/active layer/Al, respectively. The blend films showed a hole mobility of 5.8 × 10^−5^ cm^2^ V^−1^ s^−1^ and an electron mobility of 8.9 × 10^−5^ cm^2^ V^−1^ s^−1^ with balanced charge transport (*µ*_e_/*µ*_h_ = 1.53). After TA treatment, a hole mobility of 2.11 × 10^−4^ cm^2^ V^−1^ s^−1^ and an electron mobility of 2.02 × 10^−4^ cm^2^ V^−1^ s^−1^ were obtained. Apparently, after TA, charge mobility were greatly increased and exhibited a more balanced charge transport (*µ*_h_/*µ*_e_ = 1.04), which probably TA can induce microphase separation and highly ordered interpenetrating networks. The improved *J*_sc_ and FF with TA could be attributed from the higher and more balanced charge carrier mobilities in the HFQx‐T:m‐ITIC‐OR blend films.^47^, ^48^

**Table 3:** The mobilities data and power‐law exponent of PSCs based on HFQx‐T:m‐ITIC‐OR blend films

Active layer	Ratio	*µ*_h_ [cm^2^ V^−1^ s^−1^]	*µ*_e_ [cm^2^ V^−1^ s^−1^]	*µ*_e_/*µ*_h_	αc)
HFQx‐T:m‐ITIC‐OR	1:1a)	5.8 × 10^−5^	8.9 × 10^−5^	1.53	0.94
	1:1b)	2.11 × 10^−4^	2.02 × 10^−4^	1.04	0.98

^a)^ Without annealing

^b)^ Annealing at 150 °C for 5 min

^c)^ Power‐law exponent.

Besides the high and balanced carrier mobility, the other factor of affecting photovoltaic performance is charge recombination behavior in the device. Light intensity (*P*)‐dependent photocurrent measurements were carried out to gain insight into the charge recombination behavior in the devices. Generally, the correlation of *J*_sc_ and *P* can be described as *J*_sc_ ∝ P^α^. If the bimolecular recombination of the free carriers is negligible, the power‐law exponent (α) should be equal to 1.^49^, ^50^ As shown in Figure 4c, the values of α for HFQx‐T:m‐ITIC‐OR blend films without TA was 0.94, which indicated there was still some bimolecular recombination. However, the α increased to 0.98 after TA, which suggested efficient charge transport and weaker biomolecular recombination. The suppressed charge recombination agrees well with the higher FF of 0.64 and higher *J*_sc_ of 16.15 mA cm^−2^.

**Figure 5:**
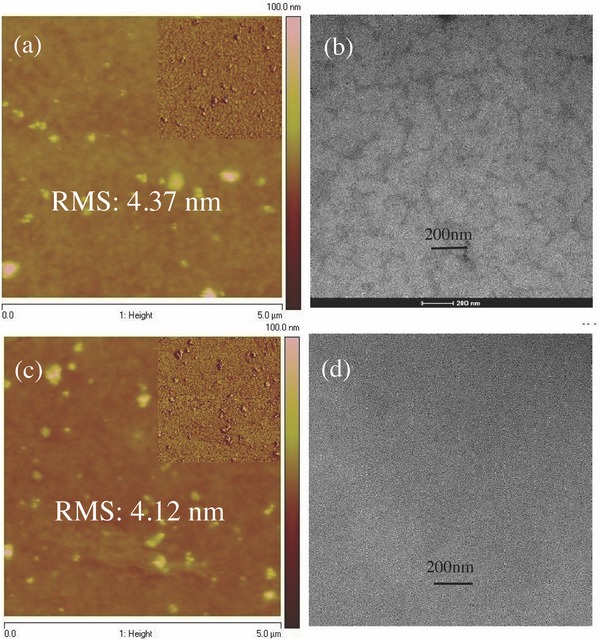
Tapping‐mode AFM images of HFQx‐T:m‐ITIC‐OR (1:1, w/w) blend films a) without TA and c) with TA; the insets show corresponding AFM phase images. TEM image of the HFQx‐T:m‐ITIC‐OR (1:1, w/w) blend films b) without TA and d) with TA.

Tapping‐mode atomic force microscopy (AFM) was used to investigate the surface morphology of the optimized photoactive layer as shown in **Figure** **5**a,c, the surface of HFQx‐T:m‐ITIC‐OR at (1:1, w/w) blend film were relatively rough with a root‐mean‐square (RMS) roughness of 4.37 and 4.12 nm without and with TA, respectively. There is only slight change of RMS roughness with or without TA. Furthermore, we investigated the internal morphology of HFQx‐T:m‐ITIC‐OR blend films by transmission electron microscopy (TEM). As shown in Figure 5b, without TA, little large phase separation and amorphous regions in the HFQx‐T:m‐ITIC‐OR blend films could be observed. In contrast, very homogeneous and interpenetrating networks with small crystalline grains uniformly covering the entire scan area can be seen only after TA (Figure 5d). Large crystalline domains is easy to get recombination for photogenerated excitons before reaching the interfaces of the donor and acceptor, which is harmful for *J*_sc_ and FF.^51^ The results indicated that changes of the interior morphology after TA treatment could be favorable for efficient exciton dissociations in the device, thus giving rise to an increased *J*_sc_ and FF.^51^, ^52^

## 3 Conclusion

A promising electron acceptor m‐ITIC‐OR with *meta*‐alkoxyphenyl side chains was first designed and synthesized, showing broad absorption and matched energy levels with HFQx‐T. The optimized HFQx‐T:m‐ITIC‐OR blend films possess complementary absorption, high and balanced charge transport, as well as nanoscale interpenetrating morphology, which contributed to high performance in PSCs. Particularly, after TA, *µ*_e_/*µ*_h_ reached a very balanced value of 1.04, which is beneficial for high FF and *J*_sc_. The nonfullerene PSCs based on HFQx‐T:m‐ITIC‐OR (1:1, w/w) blend films exhibited an impressive PCE of 9.3% with a *V*_oc_ of 0.90 V, a *J*_sc_ of 16.07 mA cm^−2^, and a FF of 0.64 only after TA. This efficiency is among one of the highest PCEs in additive‐free PSCs. Our preliminary investigations demonstrate that *meta*‐alkoxyphenyl side chains are very promising for constructing high‐performance acceptor materials. This is a new electron acceptor with *meta*‐alkoxyphenyl side chains with an efficiency of over 9%. Despite high PCEs from HFQx‐T:m‐ITIC‐OR blend films in this work, we strongly believe that there is still much space to further improve photovoltaic performance by material modification and device engineering.

## Conflict of Interest

The authors declare no conflict of interest.

## Supporting information

Supplementary
