# Supplementary material for: A New Electron Acceptor with meta‐Alkoxyphenyl Side Chain for Fullerene‐Free Polymer Solar Cells with 9.3% Efficiency
Source: Adv Sci (Weinh). 2017 Aug 17;4(11):1700152. doi: 10.1002/advs.201700152 (PMC5700628; doi:10.1002/advs.201700152)
Supplement: Supplementary file 1 — Supplementary [file ADVS-4-na-s001.pdf]

Copyright WILEY-VCH Verlag GmbH & Co. KGaA, 69469 Weinheim, Germany, 2017.

ADVANCED SCIENCE
Open Access

Supporting Information

for *Adv. Sci.,* DOI: 10.1002/advs.201700152

A New Electron Acceptor with *meta*-Alkoxyphenyl Side Chain for Fullerene-Free Polymer Solar Cells with 9.3% Efficiency

*Zhenzhen Zhang, Liuliu Feng, Shutao Xu, Ye Liu, Hongjian Peng, Zhi-Guo Zhang, Yongfang Li, and Yingping Zou**

Supporting Information

A new electron acceptor with meta-alkoxyphenyl side chain for fullerene-free polymer solar cells with 9.3% efficiency

Zhenzhen Zhang[a+], Liuliu Feng[a+], Shutao Xu[a], Ye Liu[a], Hongjian Peng[a], Zhi-Guo Zhang[b], Yongfang Li[b], Yingping Zou[a*]

a. College of Chemistry and Chemical Engineering, Central South University, Changsha 410083, China. E-mail: yingpingzou@csu.edu.cn(Y.Zou)

b. Beijing National Laboratory for Molecular Sciences, Institute of Chemistry, Chinese Academy of Sciences, Beijing 100190, China

[+] The authors contributed this work equally.

1. **General characterization**
2. **Fabrication and characterization of polymer solar cells**
3. **Materials**
4. **Synthesis**
5. **^{1}H NMR and ^{13}C NMR**
6. **CV**
7. **All detailed photovoltaic data**

1. Characterization

^{1}H NMR and ^{13}C NMR were recorded using a Bruker DMX-400 and DMX-500 spectrometers with deuterated chloroform as solvent at 298 K. Chemical shifts were reported as δ values (ppm) with tetramethylsilane (TMS) as the internal reference. UV-Vis absorption spectra were measured with a SHIMADZU UV-2600 spectrophotometer. Cyclic voltammetry (CV) was recorded with a computer controlled CHI660E electrochemical workstation using m-ITIC-OR film on platinum electrode (1.0 cm^2) as the working electrode, a platinum wire as the counter electrode and Ag/AgCl (0.1 M) was used for the reference electrode in an anhydrous and argon-saturated solution of 0.1 M of tetrabutylammonium hexafluorophosphate (Bu_4NPF_6) in acetonitrile at a scanning rate of 50 $mV\cdot s^{-1}$. PL spectra were measured with a Hitachi F-4600 fluorescence spectrophotometer. The morphologies of the HFQx-T: m-ITIC-OR blend films were investigated by atomic force microscopy (AFM, Agilent Technologies, 5500 AFM/SPM System, USA) in contacting mode with a 5 μm scanner. Transmission electron microscope (TEM) measurements were performed in a JEM-2100F.

2. Fabrication and characterization of polymer solar cells.

The non-fullerene PSCs were fabricated with a configuration of

ITO/PEDOT:PSS/active layer/PDINO/Al. A thin layer of PEDOT:PSS was deposited through spin-coating on precleaned ITO-coated glass from a PEDOT:PSS aqueous solution (Baytron P VP AI 4083 from H. C. Starck) at 4000 rpm and dried subsequently at 150 °C for 15 min in air. Then the device was transferred to a nitrogen glove box, where the active blend layer was spin-coated from chloroform solution containing a blend of HFQx-T and m-ITIC-OR onto the PEDOT:PSS layer. The optimized conditions are with a spin rate of 2800 rpm and with a blend chloroform solution containing 9 mg/mL HFQx-T and 9 mg/mL m-ITIC-OR. All blend films were thermally annealed at 150 ^{o}C for 5 min, the thickness of active layer was typically 100 nm. After cooling to room temperature, methanol solution of PDINO at a concentration of 1.0 mg/mL was deposited atop the active layer at 3000 rpm for 30 s to afford a thickness of 10 nm. Finally, top Al electrode was deposited in vacuum onto the cathode buffer layer at a pressure of ca. 8.0×10^{-5} Pa. The active area of the device was 4.5 mm^2. The current density-voltage (*J*-*V*) characteristics of the PSCs were measured in glove box on a computer-controlled Keithley 2450 Source-Measure Unit. Oriel Sol3A Class AAA Solar Simulator (model, Newport 94023A) with a 450 W xenon lamp and an air mass (AM) 1.5 filter was used as the light source. The light intensity was calibrated to 100 mW cm^{-2} by a Newport Oriel 91150V reference cell. The input photon to converted current efficiency (IPCE) was measured by

Solar Cell Spectral Response Measurement System QE-R3-011 (Enli Technology Co., Ltd., Taiwan). The light intensity at each wavelength was calibrated with a standard single-crystal Si photovoltaic cell.

3. Materials

All starting materials and reagents were obtained from J&K and Alfa Asia Chemical Co. and used without further purification. Toluene and THF were distilled from sodium/ benzophenone under nitrogen. Compound 2 and 3-(octyloxy)-1-bromobenzene were synthesized according to literature procedures.[1,2]

4. Synthesis

Compound 3

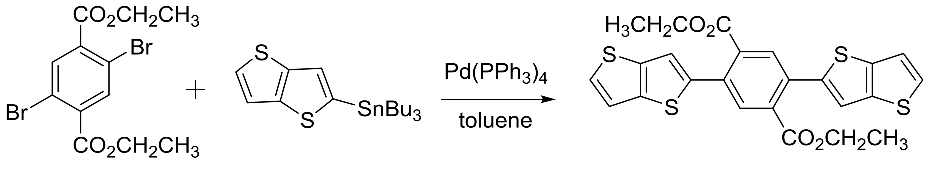

1 2 3

To a solution of compound 1 (0.43 g, 1.12 mmol) and compound 2 (1.06 g, 2.46 mmol) in anhydrous toluene (10 mL), $Pd(PPh_3)_4$ (0.10 g, 0.09 mmol) was added under nitrogen. The resulting mixture was kept at 110 ^oC for 48 h. After cooling to room temperature, the excess solvent was

concentrated under reduced pressure and the resulting solid was washed successively with ethanol, The crude product was collected and purified by silica gel chromatography with dichloromethane/hexane (1:1 , *v/v*) as the eluent to afford 3 as yellow solid (0.46 g, 82%). ^{1}H NMR (500 MHz, $CDCl_3$) δ 7.91 (s, 2H), 7.43 (d, 2H), 7.32 (m, 4H), 4.27 (m, 4H), 1.16 (t, 6H).

Compound 4

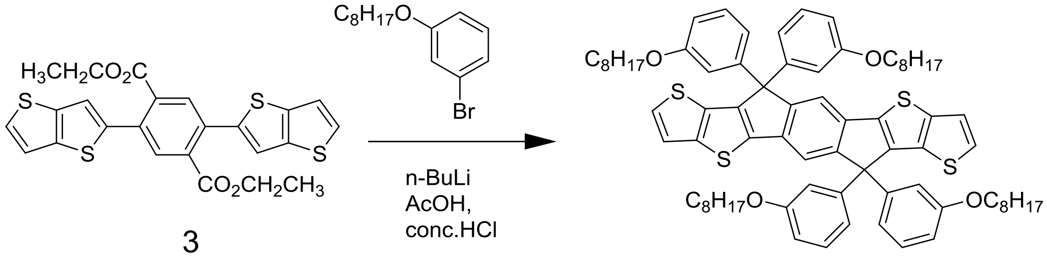

3

4

To a solution of 3-(octyloxy)-1-bromobenzene (1.55 g, 5.43 mmol) in THF (12 mL) at -78 ^{o}C was added n-BuLi (2.4 mL, 2.5 M in hexane) under argon, the mixture was kept at -78 ^{o}C for 1 h, then a solution of compound 3 (0.46 g, 0.92 mmol) in THF (50 mL) was added slowly. After the addition, the mixture was stirred at room temperature overnight and then poured into water and extracted twice with ethyl acetate. After removing the solvent, the crude product was dissolved in acetic acid (10 mL) and conc. HCl was added slowly (1 mL), then the mixture was refluxed for 4h. After cooling to room temperature ,the mixture was extracted with dichloromethane. The resulting crude compound was

purified by silica gel column using a mixture of hexane/ dichloromethane (5 : 1, *v/v*) as the eluent to give 4 as light-yellow solid (3.08 g, 28%). ^{1}H NMR (500 MHz, $CDCl_3$) δ 7.54 (s, 2H), 7.49 (d, 2H), 7.30 (d, 2H), 7.18 (m, 4H), 6.86 (m, 8H), 6.79 (m, 4H), 3.87 (t, 8H), 1.71 (t, 8H), 1.42-1.20 (m,48H), 0.89 (m, 12H).

Compound 5

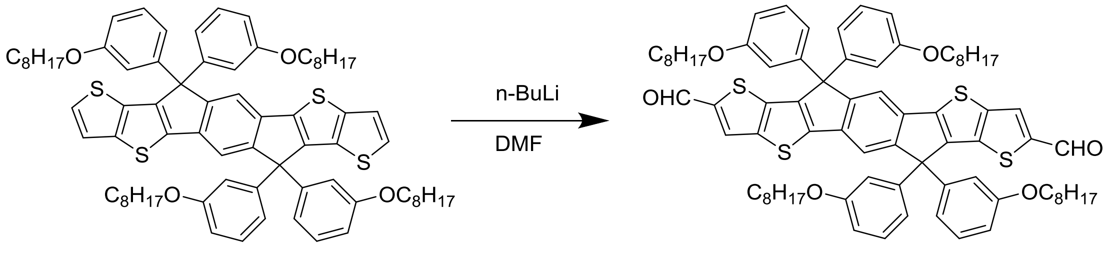

To a solution of compound 4 (3.08 g, 0.26 mmol) in anhydrous THF (30 mL) was added n-BuLi (2.5M in hexane, 0.62 mmol) dropwisely at -78 ^{o}C under argon. The reaction mixture was stirred at -78 ^{o}C for 1 h, and then anhydrous DMF (0.06 mL) was added. The mixture was stirred overnight at room temperature. Brine was added and the mixture was extracted with dichloromethane. The organic phase was evaporated and the residue was purified by silica gel column using a mixture of hexane/ dichloromethane (1 : 1, *v/v*) as the eluent to give 5 as orange solid (0.23 g, 70%). ^{1}H NMR (500 MHz, $CDCl_3$) δ 9.91 (s, 2H), 7.96 (s, 2H), 7.65 (s, 2H), 7.23(t, 4H), 6.83 (m, 12H), 3.88 (t, 8H), 1.73-1.42 (m, 48H), 0.90 (m, 12H).

Synthesis of m-ITIC-OR

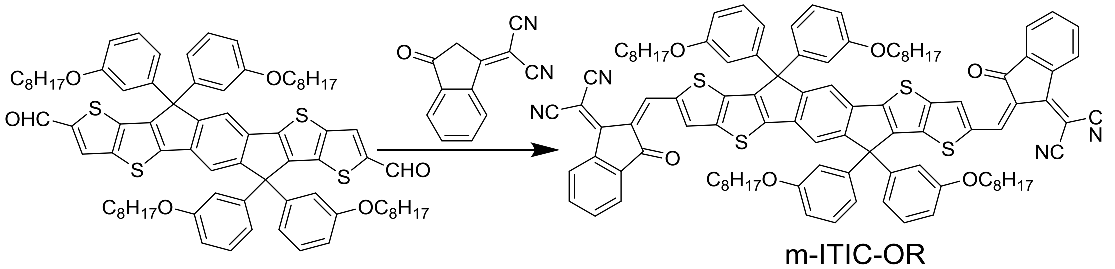

5

m-ITIC-OR

To a solution of compound 5 (0.23 g, 0.18 mmol) and IC (0.35 g, 1.81 mmol) in $CHCl_3$ (70 mL) was added pyridine (1.5 mL) under argon, and then was refluxed for 16 h . After cooling to room temperature, the mixture was poured into methanol and filtered. The residue was purified by column chromatography using hexane/ dichloromethane (1:1) as eluent, yielding m-ITIC-OR as dark blue solid (150 mg, 52%). ^{1}H NMR (500 MHz, $CDCl_3$) δ 8.91 (s, 2H), 8.71 (d, 2H), 8.17(s, 2H), 7.88(d, 2H), 7.76(m, 4H), 7.68 (s, 2H), 7.26(t, 4H), 6.87 (m, 12H), 4.01 (m, 8H), 1.52-1.98 (m, 48H), 0.88 (m, 8H). ^{13}C NMR (125 MHz, $CDCl_3$) δ 188.02, 160.31, 159.49, 154.93, 153.07, 147.01, 146.94, 143.55, 143.04, 140.05, 139.60, 138.24, 136.92, 136.88, 135.18, 134.43, 129.83, 125.35, 123.63, 122.71, 120.02, 118.76, 114.78, 114.61, 114.54, 113.64, 77.28, 77.03, 76.78, 69.48, 31.83, 31.60, 29.45, 29.07, 26.93, 25.29, 11.44. Anal.: Calc. for $C_{102}H_{98}N_4O_6S_4$: C, 76.37; H, 6.16; N, 3.49. Found: C, 76.13; H, 5.97; N, 3.42%.

5. ^{1}H NMR and ^{13}C NMR

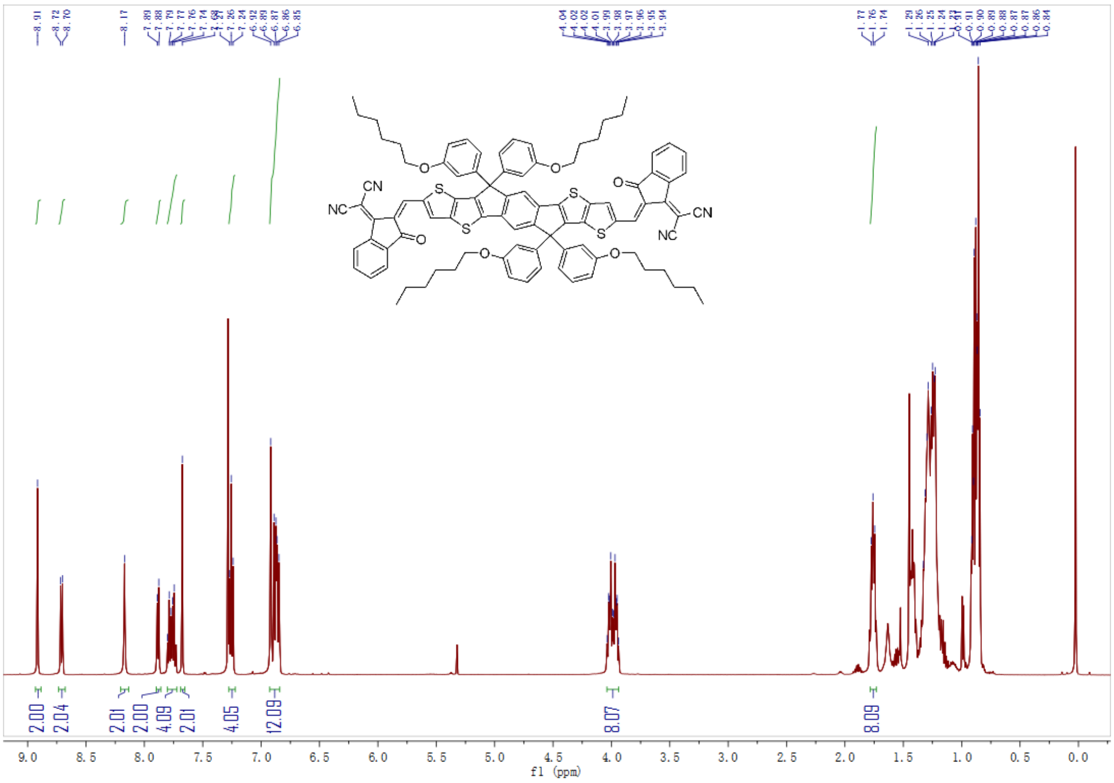

Figure S1. ^{1}H NMR spectrum of m-ITIC-OR.

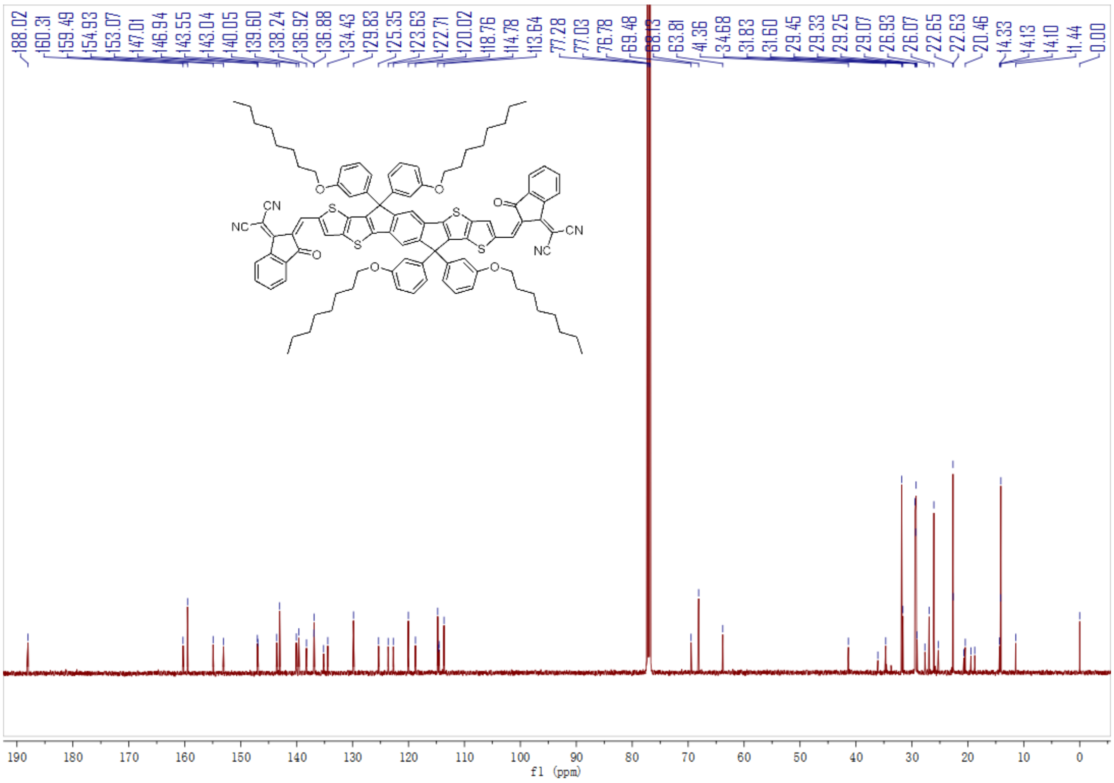

Figure S2. ^{13}C NMR spectrum of m-ITIC-OR.

6. CV

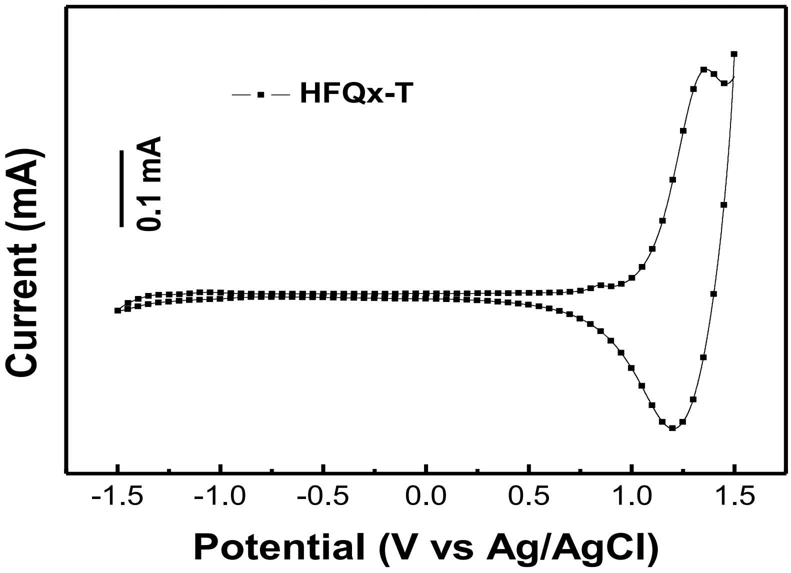

Figure S3. Cyclic voltammogram of HFQx-T in CH_3CN/0.1M Bu_4NPF_6.

7. All detailed photovoltaic data

Table S1. Device parameters of PSCs based on HFQx-T: m-ITIC-OR under illumination of AM 1.5G,100 mW cm^{-2}.

Active layer	Ratio	V_{oc} (V)	J_{sc} (mA/cm^2)	FF (%)	PCE (%)	Average PCE(%)[c]
	1:1[a]	0.95	13.48	50	6.36	6.28
	1:1[b]	0.90	16.15	64	9.30	9.13
HFQx-T:	1:1.5 [a]	0.95	13.27	55	6.93	6.85
m-ITIC-OR	1:1.5 [b]	0.89	15.08	68	9.23	9.11
	1.5:1 [a]	0.95	12.53	46	5.33	5.15
	1.5:1 [b]	0.86	14.68	55	6.96	6.83
HFQx-T：	1:1 [a]	0.95	13.42	62	7.99	7.65
ITIC	1:1 [b]	0.90	14.89	66	9.07	9.06

[a] Without annealing. [b] Annealing at 150 ^{o}C for 5 min. [c] average PCEs are from 10 devices.

Reference

[1] K. Kawabata, M. Takeguchi and H. Goto, *Macromolecules*, **2013**, *46*, 2078.

[2] M. Wakioka, M. Ikegami and F. Ozawa, *Macromolecules*, **2010**, *43*, 6980.
